# Food and Aeroallergen Sensitization in IgE -Mediated Asthma in Egypt

**DOI:** 10.2174/1874306402115010052

**Published:** 2021-12-31

**Authors:** Abdellah H.K. Ali

**Affiliations:** 1 Department of Chest Diseases and Tuberculosis, Sohag Faculty of Medicine, Sohag University, Sohag, Egypt

**Keywords:** Atopic asthma, Allergens, Polysensitization, Antibodies, Antigen, Adverse reactions

## Abstract

**Purpose:**

Identifying the distribution of allergens is valuable to the effective diagnosis and treatment of allergic disease. So, our aim is to explore the sensitization of food and aeroallergens in Egyptian patients with atopic asthma.

**Methods:**

Cross-sectional study recruited 268 Egyptian patients with atopic asthma. Asthmatic patients were assessed by the enzyme allegro sorbent test (EAST) method for specific IgE to a panel of 19 common regional inhaled allergens and 15 food allergens.

**Results and Discussion:**

One hundred percent of the patients were sensitive to at least one allergen. Allergy to food allergens only was 2.9%; inhaled allergens only were 26.2% and both were70.9%. Fungi (62%) were the most frequent sensitizing aeroallergen amongst our asthmatic patients, followed by the pollen allergens (42.5%) and house dust mites (HDMs) (26%). Cows’ milk (30.5%) was the most frequent sensitizing food amongst our asthmatic patients, followed by eggs (22.4%) and fish (21.6%). Mono-sensitized patients accounted for 6.7% of all cases, while polysensitized was 93.3%. Moderate and severe asthma showed a significantly higher frequency of polysensitization compared to mild asthma.

**Conclusion:**

Fungi and cow's milk are the chief sensitizing allergens in Egyptian patients with atopic asthma. This study represents the first report of sensitization in atopic adult asthma using a large extract panel in Upper Egypt.

## 1 INTRODUCTION

Atopic asthma is an anIgE-mediated disease caused by pathological hyperresponsiveness by host immune systems to foreign allergens [1]. Allergens are antigens that induce and react with specific immunoglobulin (IgE) antibodies. Food and inhaled allergies are induced and regulated by IgE and can be present in children and adults [2]. Depending on the considerable variety of geographic location, climates, food habits and lifestyles, the types of allergens vary widely from region to region [3]. Moreover, differences in the age of the patients can produce different sensitive allergens [4, 5].

Many in vitro and in vivo laboratory tests are used for the diagnosis of allergic diseases. Measuring IgE antibodies in Type 1 hypersensitivity reactions is now a well-established method to identify the allergens responsible for this immune-mediated reaction which can be triggered by very small amounts of antigen [6].

The specific serum IgE assay is safe not influenced by antihistamine drugs, skin disease, or skin adverse reactions [7]. The study aims to evaluate the frequency of common allergens in atopic asthma in Sohag, Upper Egypt and to provide a reference for allergen findings in such areas.

## 2 METHODS

A one-year cross-sectional study was conducted in Allergy and Asthma outpatient Clinic, Sohag University Hospital, Sohag governorate, Egypt. Sohag has a subtropical desert dry hot climate. The study sample included asthmatic patients based on GINA criteria who were allergic with high serum total IgE according to age. Patients with high serum IgE due to immunodeficiency disease or parasite infection were excluded. This study protocol was approved by medical research committe of Sohag Faculty of medicine and was conducted in accordance with the principles of the Declaration of Helsinki. Written informed consent was obtained from the study participants prior to the study commencement. Clinical history and especially exposure to allergens and its association with clinical presentation and spirometry were made according to the standard practice. Allergic workup was applied to the selected patients who included the following tests.

a) Serum total IgE was measured by enzyme-linked immunosorbent assay (ELISA) by r-Biopharm kit, Germany. According to the results from Roche diagnostics, total serum IgE was divided into six levels depending on patient age. Reference range was: 0 - <1 yr: 0–15 IU/ml, 1- <5 yrs: 0–60 IU/ml, 5 - <9yrs: 0–90IU/ml, 9 - <15yrs: 0–200 IU/ml, 15 yrs or older: 0–150 IU/ml.

b) Serum-specific IgE for a panel of food and aeroallergens were measured by a commercially available kit (immunoblot EUROLINE, Germany) based on the enzyme allegro sorbent test (EAST), which consisted of 34 allergens. According to the recommended procedure of the manufacturer, the serum IgE results were categorized into zero to six classes according to the severity of allergic reaction to each allergen: Zero: Not or hardly present(0.00-0.34IU/ml), 1: Low threshold(0.35-0.69 IU/ml), 2:Slight increase(0.70-3.49 IU/ml), 3:Significantly increased(3.5-17.49IU/ml), 4:High(17.5-49.9IU/ml), 5:Very high(50-100IU/ml), 6:Extremely high(>100IU/ml).

The common food panel comprises the following: milk, egg, fish, shrimp banana, peanut, wheat flour, hazelnut, almond, Solanaceae, chicken, citrus mix, apple, strawberry, and cacao. The panel of aeroallergens included: Cat epithelia, dog epithelia, feather-mix, Dermatophagoides pteronyssinus, Dermatophagoides farinae, birch, sunflower seeds, mixed grasses, German cockroach, Common wasp venom, Honeybee venom, Penicillium notatum, Aspergillus Niger, Aspergillus fumigatus, Candida albicans, Alternaria alternata, Trichophyton mentagrophytes, latex, and wool.

### 2.1 Statistical Methods

The statistical analysis was performed using SPSS (version 20). Data are expressed as the means and standard deviations (SDs) for continuous variables and frequencies and percentages for categorical variables. The Z score test for two population proportions is used. The results were considered significant if *P* ≤ 0.05.

## 3 RESULTS

In the current study, a total of 268 patients (43.2% males and 56.7%. females) mean age was 28.37±19.58years. Concomitant allergic rhinitis was diagnosed in 43 patients (16.04%) of the studied group. Mean serum total IgE level was 312.06±218.30. A family history of bronchial asthma was reported in 76 patients (28.35%), as shown in Table **1**.

**Fig. (1):**
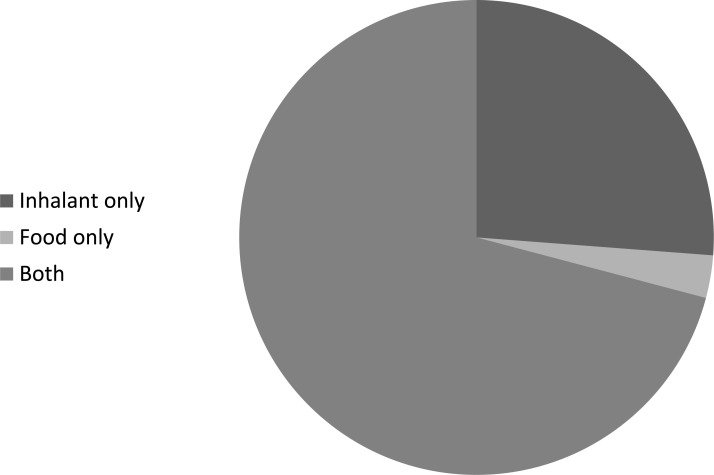
Frequency of aeroallergens and food allergens among atopic asthma.

**Fig. (2):**
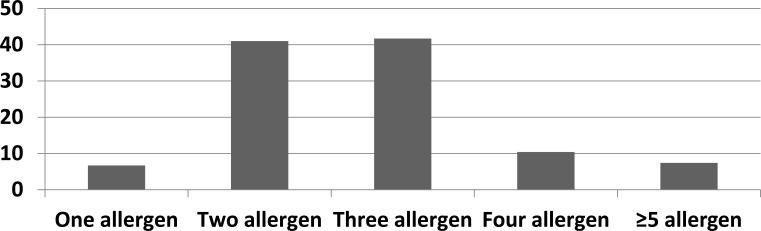
The number of allergens among sensitized patients.

One hundred percent of the patients were sensitive to at least one allergen. 2.9% of patients had positive test results for only food allergens, 26.2% of patients for only inhalant allergens and 70.9% of patients for both inhalant and food allergen (Fig. **1**). 6.7% were positive for one aeroallergen, 41% were positive for two allergens, 41.7% were positive for three allergens, 10.4% were positive for four allergens and 7.4% were positive for ≥ 5 allergens. Mono-sensitization was accounted for 6.7% of all cases; while polysensitization was 93.3% (Fig. **2**). The prevalence of aeroallergens was more than food allergens. Aeroallergens were detected in 260(97%) of all patients while food allergy was detected in 198 (73.8%) of all cases.

**Fig. (3):**
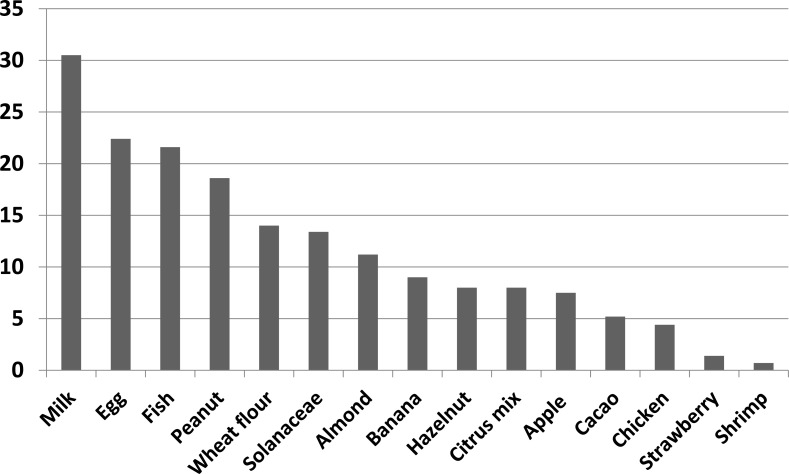
Prevalence of each type of food allergens in the studied sample.

Among food allergens, Cow's milk (30.5%) followed by eggs (22.4%) and fish (21.6%) were the most frequent sensitizing foods in our asthmatic patients. Other food allergens were peanut, wheat, Solanaceae, almond, banana, hazelnut, citrus-mix, apple and cocao (Fig. **3**). In addition, among these allergens, Solanaceae (31.24 IU/ml) followed by wheat (6.36IU/mL) and almond (5.37IU/mL) had the highest antibody titer; respectively. Titers of different food allergens are shown in Table **2**.

**Fig. (4):**
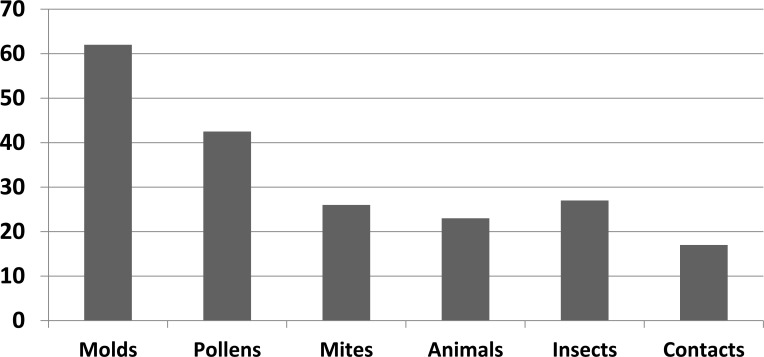
Frequency of sensitization to different groups of aeroallergens.

Among inhaled allergens, Fungi (62%), followed by pollens (42.5%) and HDMs (26%), were the most frequent sensitizing aeroallergens in our asthmatic patients (Fig. **4**). Candida albicans and aspergillus fumigates and alternarias alternate were the most common sensitizing molds (18.6%,17.9%, and 15.6%, respectively). As regards HDMs, Dermatophagoides pteronyssinus and Dermatophagoides farinae were positive among 13.4% and 12.6% of patients, respectively. With respect to animal dandruff, dog epithelia followed by cat epithelia were positive in 6% and 8.2% of patients, respectively. Moreover, sensitization to the feathers of birds was 9% (Table **3**). In addition, among aeroallergens, alternaria alternate (18.8IU/mL) followed by Sunflower seed pollens (12.73IU/mL) and Trichophyton mentagrophytes (10.07IU/mL) had respectively the highest antibody titer. Frequency and titers of specific IgE to aeroallergens inpatient with atopic asthma are displayed in Table **3**.

In Table **4**, based on the frequency of sensitization among asthmatic patients, we compared the severity of asthma with the frequency of sensitization. Moderate and severe asthma showed a significantly higher polysensitization frequency compared to mild asthma (P<0.0001).

## 4 DISCUSSION

This study was conducted to determine the prevalence of food and aeroallergens sensitization in atopic asthma in adults. We studied 34 common sensitizing inhalants and food allergens using Specific IgE assay in sera from 268 atopic asthma. This is the first study to investigate both food and aeroallergens distribution in adult atopic asthma in Sohag city, a major agricultural district, Upper Egypt.

Taking the sample, one hundred percent of atopic asthma patients were sensitized to at least one of the studied 34 allergens by utilizing SIgE assay. The sensitization to food allergens was detected in 198 (73.8%) of all cases while the sensitization to aeroallergens were in 260 (97%) of all patients. Compared to our study, the results of the previous studies demonstrated a range of sensitivity from 50 to 80 percent to at least one allergen [8-11]. A Brazilian study demonstrated that among the atopic patients, sensitization to inhaled and/or food allergen was significantly higher, oscillating between 63.6% and 98.2% [12].

Studies concerning the prevalence of food allergy among adult atopic asthma are lacking in Egypt. We examined 15 major food allergens. Here, the highest sensitivity to food allergens was Cow's milk, followed by eggs, fish, and peanut and wheat flour, respectively. Animal proteins included in our research were the most sensitizing food allergens. Sensitization to major food allergens in Sohag was compatible with worldwide literature [13-16]. Approximately similar results were reported in two of Egypt’s Arab neighboring countries: in the city of Doha and Makka [17, 18]. However, our findings are in contrast with the findings of Nabavi and colleagues [19] and Kumar *et al*. [20]. Differences in dietary habits, age and race of the patients can produce different prevalence of food allergens in various studies. It was found that cow's milk, egg white and peanut allergy were related to increased steroid use and increased hospitalization in patients with asthma [21].

In addition to food allergens, aeroallergens are the other important factors in the development and exacerbation of respiratory allergic diseases. There is remarkable variability in aeroallergen distribution among countries and even among regions within the same country [22]. Fungi (62%) were the most frequent sensitizing aeroallergen among inhaled allergens in our asthmatic patients. Candida albicans was the most common sensitizing mold (18.6%). In the north of Egypt, the fungal sensitization rate was 15.57%, in which the most common mold was Candida albicans (12.3%) [23]. In another study, Khatab *et al*. found at least one fungal allergens sensitization was 41.9% [24]. In Iran, sensitization to fungal allergens was reported in 23.7% of the patients [25]. Sathavahana Chowdary *et al*. and O’Driscoll *et al*. reported that sensitivity to fungi was 44% and 66%, respectively [26, 27].

Our population exhibited a sensitization rate of 42.5% to pollens in atopic asthma. The common pollens to which allergies were seen were birch, sunflower seeds, and mixed grasses. In Egypt, Hosney *et al*. found that the frequency of sensitization to Timothy grass pollens in males in series was 56%, while in females, it was 70.6%, with no significant difference in between [28]. Pollens sensitizations were the most representative aeroallergen sensitization (44.9%) in Portugal and in southwest Germany [29, 30]. Nevertheless, it was 87.1% in Kuwait [31]. In Saudi Arabia, 61% of the entire cohort was sensitized to at least one type of weed pollen [32]. Climate, temperature, and herbal geography are responsible for the variation [33].

The present study demonstrated a 26% prevalence rate of positive sIgE to HDMs (13.4% DP and 12.6% to DF) in patients with allergic asthma. In the north of Egypt, Hossny *et al*. found that the sensitization rate to HDMs in asthmatic children was 24% and Dermatophagoides farina and Dermatophagoides pteronyssinus were the most common, causing sensitization in 11% and 12% of patients, respectively [34]. In the KSA and UAE, the rate of sensitization to HDMs was 27% and 46%, respectively. It was much higher in warm, humid subtropical areas such as Taiwan (85%), South India (89.7%), and Brazil (73.5%) [35].

Furthermore, our data clearly demonstrated that 6.7% of the patients were mono-sensitized patients and 93.3% were polysensitized. Moderate and severe asthma showed a significantly higher polysensitization frequency compared to mild asthma (P<0.0001). Many studies revealed a higher frequency of polysensitization in patients with respiratory allergy [36, 37]. In accordance with the literature, Ciprandi *et al*., found that polysensitization was associated with more severe clinical manifestation [38]. Another study by Valero *et al*. found that the mean number of allergens in sensitized patients was 6.5 ± 2 [39]. In a study from USA, three or more allergens were positive in 81%of mild-to-moderate asthma patients [40]. According to these results, polysensitization is common among allergic patients but the rate is different depending on the region and the population.

## CONCLUSION

Our study is the first in Egypt that has evaluated sensitization patterns to food and inhaled allergens in bronchial asthma. Identifying the distribution and abundance of allergens in our region could be so helpful in controlling bronchial asthma. Animal proteins included in our research were the most sensitizing food allergens. On the other hand, Fungi followed by pollens and HDMs were the most sensitizing aeroallergens. Furthermore, moderate and severe asthma showed a significantly higher frequency of polysensitization compared to mild asthma. Based on patterns of sensitization, cost-effective specific IgE panels for screening atopic asthma patients can be provided. Further research on specific allergen immunotherapy in these populations is needed.

## Figures and Tables

**Table 1:** Clinical and laboratory data of the patients.

Age	28.37±19.58
**Sex(M/F)**	116/152
**Type of Asthma** Mild Moderate Severe	21 (7.83%) 149 (55.60%) 98(36.57%)
**Comorbidities** Allergic rhinitis Allergic conjunctivitis Atopic dermatitis	43(16.04%) 19(7.08%) 21(7.83%)
**Family History of Asthma**	76 (28.35%)
Total IgE	312.06±218.30
**Spirometry** FEV_1_ FVC FEV_1_/FVC	53.94±21.25 64.04±17.60 67.77±16.69

**Table 2:** Frequency and Titers of specific IgE to foods in patient with atopic asthma.

Allergens	Frequency	SIgETiter	Class
Milk	82(30.5%)	2.71	2
Egg	60(22.4%)	1.77	2
Fish	58(21.6%)	1.79	2
Peanut	50(18.6%)	3.59	3
Wheat flour	38(14%)	6.36	3
Solanaceae	36(13.4%)	31.24	4
Almond	30(11.2%)	5.37	3
Banana	24(9%)	0.51	1
Hazelnut	22(8%)	1.94	2
Citrus mix	22(8%)	3.05	2
Apple	20(7.5%)	1.67	2
Cacao	14(5.2%)	0.76	2
Chicken	12(4.4%)	0.78	1
Strawberry	4(1.4%)	1.32	2
Shrimp	2(0.7%)	0.56	1

**Table 3:** Frequency and Titers of specific IgE to aeroallergens in patient with atopic asthma.

-	Allergen	Frequency	SIgETiter	Class
Molds				
	Candida albicans	50(18.6%)	5.05	3
	Aspergillus fumigatus	48(17.9%)	2.89	2
	Alternaria alternata	42(15.6%)	18.8	4
	Trichoph mentagrophytes	16(6%)	10.07	3
	Aspergillus niger	6(2.2%)	1.92	2
	Penicillium notatum	4(1.4%)	1.77	2
Pollens				
	Birch	46(17%)	5.44	3
	Sunflower seed	38(14%)	12.73	3
	Mixed grasses	30(11.2%)	3.38	2
Animals				
	Feather-Mix	24(9%)	2.95	2
	Cat epith	22(8.2%)	4.94	3
	Dog epith	16(6%)	2.6	2
Mites				
	Derm. Pteronssinus	36(13.4%)	3.04	2
	Derm. Farinae	34(12.6%)	2.58	2
Insects				
	Cockroaches	36(13.4%)	4.18	3
	Common wasp venom	20(7.4%)	3.75	3
	Honey bee venom	16(6%)	4.6	3
Contacts				
	Latex	24(9%)	2.24	2
	Wool	22(8.2%)	2.21	2

**Table 4:** Relationship between patients with atopic asthma and degree of sensitization.

Degree of Asthma	Type of Sensitization		
	Mono-Sensitization, n = 18 (%)	Poly-Sensitization, n =250 (%)	P Value
Mild (intermittent-persistent)	13(61.90)	8 (38.10)	<0.00001
Moderate	4 (2.68)	145(97.32)	<0.00001
Severe	1 (1.03)	97 (98.97)	<0.00001

## Data Availability

None.
